# Room-temperature spin injection from a ferromagnetic semiconductor

**DOI:** 10.1038/s41598-023-29169-9

**Published:** 2023-02-07

**Authors:** Shobhit Goel, Nguyen Huynh Duy Khang, Yuki Osada, Le Duc Anh, Pham Nam Hai, Masaaki Tanaka

**Affiliations:** 1 Department of Electrical Engineering and Information Systems, The University of Tokyo, 7-3-1 Hongo, Bunkyo-ku, Tokyo, 113-8656 Japan; 2 CREST, Japan Science and Technology Agency, 4-1-8 Honcho, Kawaguchi, Saitama 332-0012 Japan; 3 Department of Electrical and Electronic Engineering, Tokyo Institute of Technology, 2-12-1 Ookayama, Meguro-ku, Tokyo, 152-8550 Japan; 4 Department of Physics, Ho Chi Minh City University of Education, 280 An Duong Vuong Street, District 5, Ho Chi Minh City, 738242 Vietnam; 5 Institute of Engineering Innovation, The University of Tokyo, 7-3-1 Hongo, Bunkyo-ku, Tokyo, 113-8656 Japan; 6 PRESTO, Japan Science and Technology Agency, 4-1-8 Honcho, Kawaguchi, Saitama 332-0012 Japan; 7 Center for Spintronics Research Network (CSRN), The University of Tokyo, 7-3-1 Hongo, Bunkyo-ku, Tokyo, 113-8656 Japan; 8 Institute for Nano Quantum Information Electronics, The University of Tokyo, 4-6-1 Komaba, Meguro-ku, Tokyo, 153-8505 Japan

**Keywords:** Spintronics, Spintronics

## Abstract

Spin injection using ferromagnetic semiconductors at room temperature is a building block for the realization of spin-functional semiconductor devices. Nevertheless, this has been very challenging due to the lack of reliable room-temperature ferromagnetism in well-known group IV and III-V based semiconductors. Here, we demonstrate room-temperature spin injection by using spin pumping in a BiSb/(Ga,Fe)Sb heterostructure, where (Ga,Fe)Sb is a ferromagnetic semiconductor (FMS) with high Curie temperature (*T*_C_) and BiSb is a topological insulator (TI). Despite the very small magnetization of (Ga,Fe)Sb at room temperature (45 emu/cc), we detected spin injection from (Ga,Fe)Sb by utilizing the large inverse spin Hall effect (ISHE) in BiSb. Our study provides the first demonstration of spin injection at room temperature from a FMS.

## Introduction

Room temperature injection and detection of spin-polarized carriers from ferromagnetic semiconductors (FMSs), which possess both the properties of ferromagnets and semiconductors, are crucial for realizing future semiconductor-based spintronics devices^1,2^. Indeed, one of the 125 big questions raised about two decades ago was, “*Is it possible to create magnetic semiconductors that work at room temperature?*”^3^ So far, all room-temperature spin injection and detection experiments use ferromagnetic metals because of their high Curie temperature (*T*_C_). However, ferromagnetic metals suffer from the “conductivity mismatch” problem when used in heterojunctions with semiconductors, resulting in very low spin injection/detection efficiencies in semiconductor spintronics devices^4,5^. Thus, it is crucial to develop ferromagnetic semiconductors with high *T*_C_ and room-temperature spin injection capability. Meanwhile, successful spin injection has been reported only in prototypical Mn-doped III-V FMS (Ga,Mn)As at very low temperature < 120 K^6–8^. This is because these Mn-doped FMSs show ferromagnetic order only at low temperature (the maximum Curie temperature *T*_C_ is 200 K in (Ga,Mn)As)^9^. Recently, Fe-doped III-V FMSs, such as (In,Fe)As, (Ga,Fe)Sb, and (In,Fe)Sb, are shown to be promising because of their high *T*_C_ (> 300 K)^10–17^. The origin of ferromagnetism as well as the electronic structures and magnetic properties of Fe-doped FMSs have also been studied by first-principles calculations^18,19^, which suggest that the ferromagnetic interaction between Fe atoms occurs at the second nearest-neighbor and third nearest-neighbor in the semiconductor hosts. It is also shown that high *T*_C_ in Fe-doped FMSs may come from high impurity concentration and is not due to the carrier-induced mechanism because Fe^3+^ does not introduce carriers^20^. Furthermore, *T*_C_ over 1000 K for Fe-doped FMSs is predicted, based on *n*- or *p*-type Fermi level shift^21^. Thus, Fe-doped FMSs are very promising for future applications because high Curie temperature (*T*_C_ > 500 K) is necessary for practical applications^22^. Very recently, a giant magnetoresistance over 500% was observed in *n*-type (In,Fe)As/*p*-type (Ga,Fe)Sb spin diodes at low temperatures^23^. Despite all of these intensive efforts, room-temperature spin injection from Fe-doped FMSs has not yet been demonstrated.

Recently, we show macroscopic ferromagnetism in (Ga,Fe)Sb by observing clear ferromagnetic resonance (FMR) at room temperature^12,13^. So far, spin pumping^24,25^, electrical spin injection^26,27^, spin Seebeck effect^28^, and other optical methods^29^ have been used to study the spin injection from a ferromagnetic (FM) layer into a non-magnetic (NM) layer. Among those methods, spin pumping, which employs FMR, is one of the most efficient methods, because a pure spin current can be transported through a FM/NM interface even under a conductivity mismatch condition^30^. The injected spin current is detected by the inverse spin Hall effect (ISHE) in the NM layer, which converts the spin current to a charge current via spin–orbit interaction (SOI)^31^. The efficiency of this spin-to-charge conversion is characterized by the inverse spin Hall angle (ISHA). However, because the ISHE output voltage is proportional to the magnetization of the FM layer, which is only 45 emu/cc for (Ga,Fe)Sb at room temperature, one needs to use a spin Hall material with a large spin Hall effect as the spin detector. From this viewpoint, topological insulators can be utilized to detect spin injection from the (Ga,Fe)Sb^32,33^. Recently, it was found that Bi_1-*x*_Sb_*x*_ (0.07 ≤ *x* ≤ 0.22) is a three-dimensional topological insulator (TI) with strong SOI and large spin Hall angle (SHA) > 1^34^. This large SHA was also observed in non-epitaxial BiSb layers deposited by magnetron sputtering^35–39^, which means that poly-crystalline BiSb can also be used for spin detection by using its large SHA. Non-trivial topologically protected surface states of BiSb have been confirmed by angle-resolved photoemission spectroscopy (ARPES)^40–42^, quantum oscillations^43,44^, resistivity-temperature characteristics^45,46^, and very recently, scanning tunneling microscopy (STM) measurements^47^.

In this work, we prepare a BiSb/(Ga,Fe)Sb heterostructure, where we use (Ga,Fe)Sb (*T*_C_ > 300 K) as a FMS layer and BiSb as the NM layer, and demonstrate spin injection by spin pumping and spin detection by ISHE at room temperature. Furthermore, by carefully analyzing the voltage signals at various magnetic field directions, temperatures, and microwave powers, we distinguish the intrinsic ISHE signals from parasitic galvanomagnetic contributions, such as anomalous Hall effect (AHE) and planar Hall effect (PHE)^8,48,49^. Our study provides an important step towards realization of room-temperature semiconductor spintronics devices, such as spin diodes and spin transistors.

## Results

### Sample growth and experimental procedure

**Figure 1:**
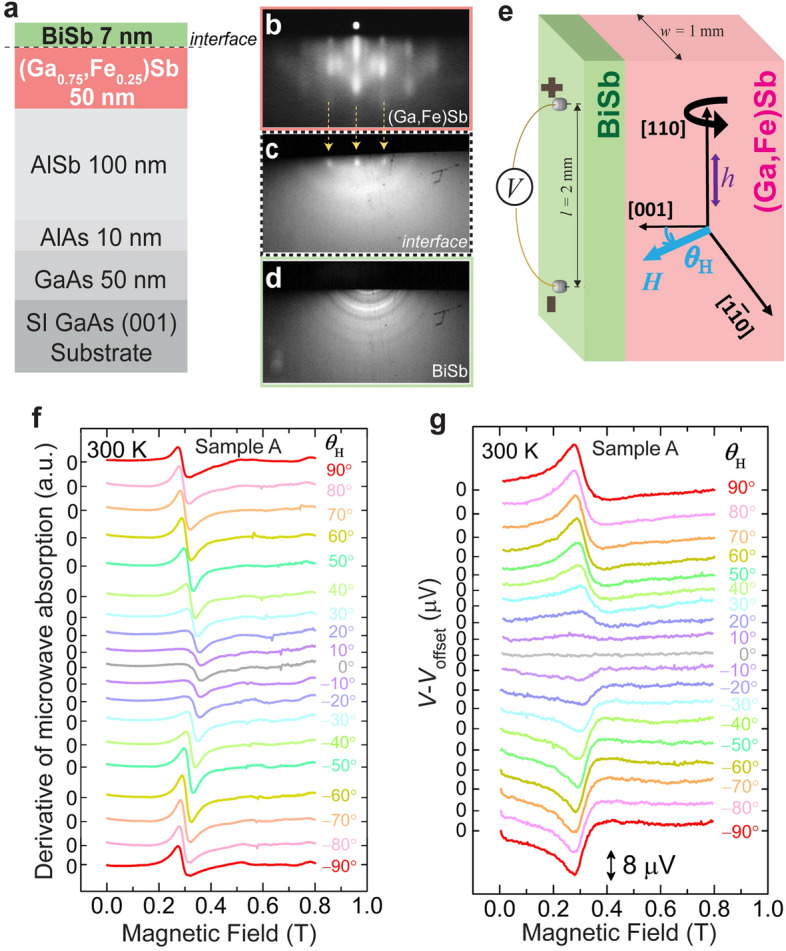
(**a**) Schematic illustration of the (001)-oriented sample structure composed of BiSb (7 nm)/(Ga,Fe)Sb (50 nm)/AlSb (100 nm)/AlAs (10 nm)/GaAs (50 nm) grown on a semi-insulating (SI) GaAs (001) substrate. (**b**–**d**) In-situ reflection high energy electron diffraction (RHEED) patterns observed along the $[\overline{1}{\text{1}}{{0}}]$ axis with a streaky RHEED pattern during the MBE growth of (Ga,Fe)Sb (**b**), a spotty RHEED pattern of the interface after anti-sputtering (**c**), and a ring RHEED pattern of BiSb during sputtering (**d**). The black dotted line in (**c**) corresponds to the interface between BiSb and (Ga,Fe)Sb layer. (**e**) Sample alignment and coordinate system used in the spin pumping measurement. A microwave magnetic field *μ*_0_***h*** was applied along the [110] axis of the sample. *θ*_H_ is the angle of the magnetic field ***H*** with respect to the [001] axis, respectively, where ***H*** is in the (110) plane. (**f**) Ferromagnetic resonance (FMR) spectra and (**g**) corresponding electromotive force (EMF) voltage peaks of the BiSb/(Ga,Fe)Sb heterostructure (sample A) measured at various magnetic field ***H*** directions (*θ*_H_ = −90° to 90°) at 300 K.

Figure 1a (top panel) shows the schematic structure of our sample. In the BiSb/(Ga_0.75_,Fe_0.25_)Sb heterostructure, (Ga,Fe)Sb layer was first grown by molecular beam epitaxy (MBE) followed by BiSb growth using sputtering (see “Methods”). The growth process was monitored in situ by reflection high-energy electron diffraction (RHEED). Figure 1b–d (top panel) show RHEED patterns observed along the $[\overline{1}{\text{1}}{{0}}]$ axis of the sample. During the MBE growth, the (Ga,Fe)Sb thin film showed bright and streaky RHEED [Fig. 1b (top panel)], thereby indicating good two-dimensional growth of a zinc-blende crystal structure. After etching the GaSb cap layer, we observed spotty RHEED, indicating that the (Ga,Fe)Sb surface is exposed [Fig. 1c (top panel)]. Finally, we observed a ring RHEED pattern after depositing BiSb [Fig. 1d (top panel)], indicating poly-crystalline BiSb in our sample. In this way, we deposited a poly-crystalline BiSb film on top of the epitaxial (Ga,Fe)Sb thin film. We characterized the magnetic properties of the BiSb (7 nm)/(Ga,Fe)Sb (50 nm) heterostructure (sample A) and a reference (Ga,Fe)Sb (50 nm) thin film (sample B) using magnetic circular dichroism (MCD) spectroscopy and superconducting quantum interference device (SQUID) magnetometry (see Sections 1 and 2 in the Supplementary Information (S.I.) for detailed characterizations). From the MCD and SQUID characterizations, we confirmed the intrinsic ferromagnetism of (Ga,Fe)Sb in both samples A and B, and that (Ga,Fe)Sb is not affected by the etching of the GaSb cap layer and the BiSb overgrowth. Also, we note that 50 nm-thick (Ga_0.75_,Fe_0.25_)Sb has in-plane magnetic anisotropy at room temperature but changes to perpendicular magnetic anisotropy at low temperatures^50^. These results are further supported by magnetic-field angle dependence of the FMR spectra of (Ga,Fe)Sb in samples A and B, which indicates that there is almost no difference in magnetic anisotropy and the FMR occurs at the same resonance fields in both samples (see Section 3 in S.I. for more details).

For spin pumping measurements, we used an electron spin resonance spectrometer whose cavity resonates in the transverse electric TE_011_ mode at a microwave frequency of 9.14 GHz. Figure 1e (top panel) shows the BiSb/(Ga,Fe)Sb bilayer structure and coordinate axes used in our spin pumping experiment. For electrical measurements, we have connected the two gold wires at both edges of the sample with a distance of *l* = 2 mm apart as shown in Fig. 1e (top panel). For the measurements, a static magnetic field *μ*_0_***H*** is applied along the $[{1}\overline{\text{1}}{{0}}]$ direction in the film plane (*i.e.* *θ*_H_ = 90°), which corresponds to the easy magnetization axis of (Ga,Fe)Sb. Here, *θ*_H_ is the out-of-plane angle between ***H*** and the [001] axis. A microwave magnetic field *μ*_0_***h*** was applied along the [110] axis. The detailed measurement procedure is described in S.I.

### Room-temperature spin pumping and spin-to-charge conversion

Figure 1f (bottom panel) shows the FMR spectra at various *θ*_H_ measured at a microwave power of 200 mW. The resonance field *μ*_0_*H*_R_ of the FMR spectra changes from 290 to 336 mT when *θ*_H_ is changed from ± 90° (***H*** // $[{1}\overline{\text{1}}{{0}}]$ and [$\overline{\text{1}}{{10}}$]) to 0° (***H*** // [001]). This indicates that the 50 nm-thick (Ga_0.75_,Fe_0.25_)Sb film in this work has the in-plane magnetic anisotropy with an easy magnetization axis along the $[{1}\overline{\text{1}}{{0}}]$ axis at 300 K. Figure 1g (bottom panel) shows the electromotive force (EMF) signal *V* (offset voltage *V*_offset_ is subtracted) at various *θ*_H_. As shown in Fig. 1g (bottom panel), the *V*–*μ*_0_*H* curves exhibit voltage extrema corresponding to the resonance fields (*μ*_0_*H*_R_) in the FMR spectra, which indicates that the observed voltage signals were generated by FMR. Also, the *θ*_H_-dependence of EMF is consistent with the formula EMF ∝ ***j***_**S**_** × *σ***, where ***j***_**S**_ and ***σ*** are the spin current and spin polarization vector of the spin current, respectively^51,52^. When *θ*_H_ is changed from 90° to –90°, the voltage signal changes its sign from positive to negative, but its magnitude is the same, which cannot be explained by the Seebeck effect. The maximum EMF signal is obtained at *θ*_H_ = 90° and –90°, where ***j***_**S**_ is perpendicular to the ***σ***. On the other hand, the EMF signal disappears at *θ*_H_ = 0°, when ***j***_**S**_ is parallel to ***σ***. This indicates that the observed EMF signal is consistent with the well-established model of spin injection by spin pumping and spin-to-charge conversion by ISHE.

**Figure 2:**
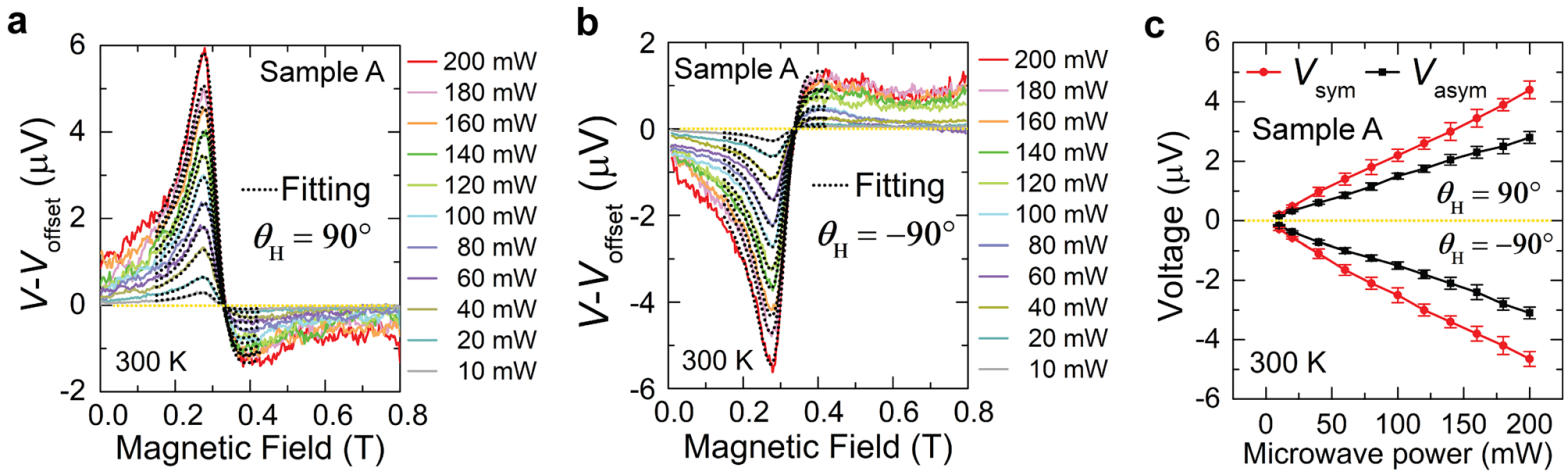
(**a**) and (**b**) Magnetic field *μ*_0_***H*** dependences of the voltage signal *V* (offset voltage *V*_offset_ is subtracted) in the BiSb/(Ga,Fe)Sb heterostructure (sample A) measured at 300 K at various microwave powers, ranging from 10 to 200 mW, for *θ*_H_ = 90° and –90°, respectively. The black dotted curves represent fitting which includes a summation of the symmetric and antisymmetric voltage components. (**c**) Microwave power dependence of the symmetric voltage component magnitude *V*_sym_ and antisymmetric voltage component magnitude *V*_asym_ at *θ*_H_ = 90° and –90°. The errors bars in (**c**) are standard deviations obtained from the fitting of the EMF peaks.

Next, we investigated EMF *vs*. magnetic field (*μ*_0_*H*) at various microwave power magnitudes to further confirm the ISHE in our sample. Figure 2a,b show the microwave power dependence of EMF for sample A at 300 K for *θ*_H_ = 90° (***H*** // [${1}\overline{\text{1}}{{0}}$]) and –90° (***H*** // [$\overline{\text{1}}{{10}}$]), respectively. We note that in both directions, the EMF is proportional to the applied microwave power, which further discards the contribution of the Seebeck effect^53^. Next, for qualitative analysis, we decomposed the obtained EMF–*μ*_0_*H* curves into a symmetric (Lorentzian) voltage component ${{V}}_{\text{sym}}^{ *}$ and an asymmetric (dispersive) voltage component ${{V}}_{\text{asym}}^{ *}$, using the following fitting expression:

1 $$\begin{aligned} \text{EMF (}{V } - \, {{V}}_{\text{offset}}) & = {{V}}_{\text{sym}}^{ *} +{{V}}_{\text{asym}}^{ *} , \\ & = {{V}}_{\text{sym}}\frac{{\left({\mu}_{0}{\Delta}{{H}}\right)}^{2}}{{\left({\mu}_{0}{{H}} \, - \, {{\mu}_{0}{{H}}}_{\text{R}}\right)}^{2}+ {\left({\mu}_{0} {\Delta}{{H}}\right)}^{2}} + {{V}}_{\text{asym}}\frac{-{2}{{\mu}}_{0} {\Delta}{{H}}{(}{\mu}_{0}{{H}} \, - \, {{\mu}_{0}{{H}}}_{\text{R}}{)}}{{\left({\mu}_{0}{{H}} \, - {{ \, {\mu}}_{0}{{H}}}_{\text{R}}\right)}^{2} +{ \left({\mu}_{0}{\Delta}{{H}}\right)}^{2}}, \end{aligned}$$

where *μ*_0_Δ*H* is the half-width at half maximum of the FMR linewidth and *μ*_0_*H*_R_ is the resonance field. Here, *V*_sym_ is the magnitude of symmetric (Lorentzian) voltage component and *V*_asym_ is the magnitude of an asymmetric (dispersive) voltage component. According to a previous work on spin pumping using (Ga,Mn)As, *V*_sym_ includes contributions from the ISHE and PHE, while *V*_asym_ originates from the AHE^8^. As shown in Fig. 2a,b, the experimental data (solid curves) is well reproduced by the fitting function (black dotted curves) of Eq. (1). From Fig. 2c, the estimated *V*_sym_ and *V*_asym_ components are directly proportional to the applied microwave power, which is an important identity of ISHE. This is also evidenced by the *θ*_H_ dependence of the EMF [Fig. 1g (bottom panel)], where we observed nearly similar voltage extrema ~ 6 μV when *θ*_H_ = 90° (***H*** // [1$\overline{\text{1}}$0]) and −90°(***H*** // [$\overline{\text{1}}$10]).

**Figure 3:**
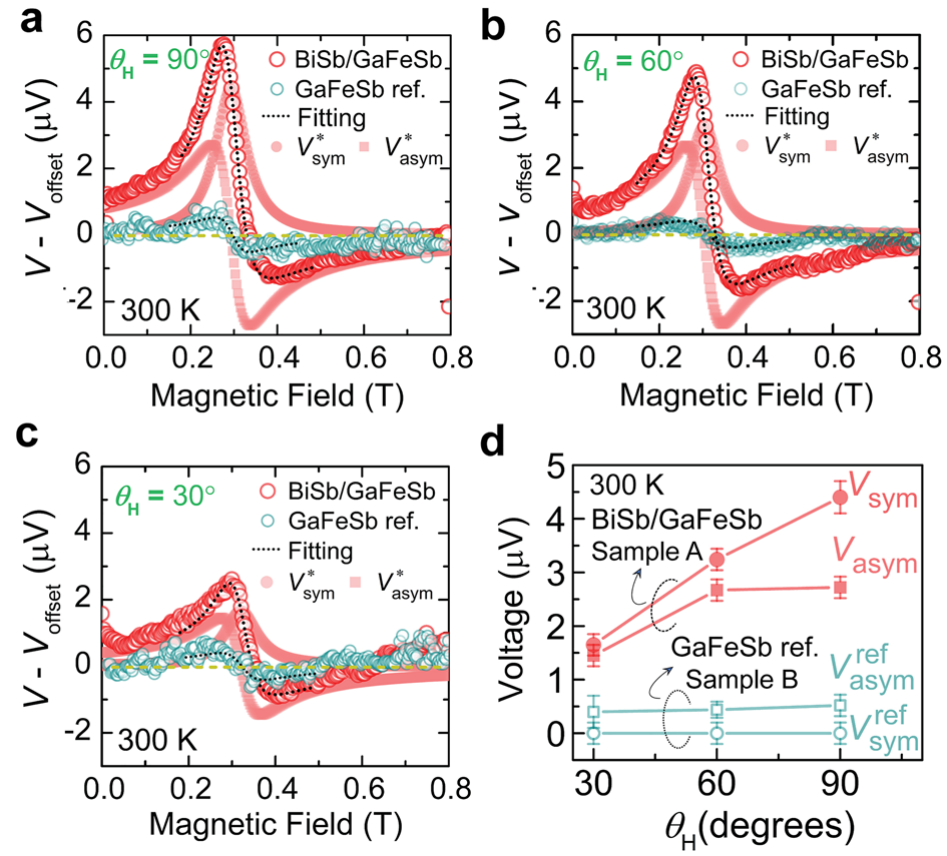
(**a**)–(**c**) Voltage signals *V*–*V*_offset_ for the BiSb/(Ga,Fe)Sb heterostructure (sample A, red big circles) and reference (Ga,Fe)Sb sample (sample B, green small circles) observed for various magnetic field ***H*** directions (*θ*_H_) at 300 K, where ***H*** is in the (110) plane. The microwave power is 200 mW. Blanked circles are experimental data, and a black color dotted line is a fitting curve. Symmetric and antisymmetric component curves, obtained from the fitting, are also shown by small solid circles and squares, respectively. (**d**) The derived symmetric voltage component magnitude (*V*_sym_) and antisymmetric voltage component magnitude (*V*_asym_) of sample A (red solid symbols) and sample B (green blank symbols) at 300 K. The circles and squares represent *V*_sym_ and *V*_asym_, respectively. The errors bars in (**d**) are standard deviations obtained from the fitting of the EMF peaks.

Next, in order to distinguish the contribution of spin-pumping to *V*_sym_ from the parasitic contribution of the PHE in sample A [BiSb/(Ga,Fe)Sb], we performed the FMR measurements on the reference sample B [(Ga,Fe)Sb without BiSb]. In Fig. 3a–c, we compare the EMF signals of sample A and sample B at various *θ*_H_. While we notice both the strong symmetric and asymmetric components in sample A, the symmetric component is absent in sample B. This result suggests that the contribution of the PHE to *V*_sym_ in (Ga,Fe)Sb is negligible. This also negates the contribution of anomalous Nernst effect (ANE) in (Ga,Fe)Sb which also has the symmetric component *V*_sym_. We then use Eq. (1) to fit to the voltage signals and estimate the magnitude of the voltage components *V*_sym_ and *V*_asym_ for sample A and ${{V}}_{\text{sym}}^{\text{ref}}$ and ${{V}}_{{\text{a}}{\text{sym}}}^{\text{ref}}$ for reference sample B. Figure 3d shows the estimated voltage components as a function of *θ*_H_. We see that ${{V}}_{\text{sym}}^{\text{ref}}$<< ${{V}}_{{\text{a}}{\text{sym}}}^{\text{ref}}$ (~ 100 times different) for sample B, which means that the voltage contribution from PHE of (Ga,Fe)Sb is negligible at 300 K. Meanwhile, the parasitic contribution from AHE of (Ga,Fe)Sb at 300 K to the asymmetric voltage component exists in both samples. However, in the case of sample A, *V*_sym_ > *V*_asym_, which means that ISHE is stronger and dominant over AHE.

### Temperature dependence of the spin injection by spin-pumping

**Figure 4:**
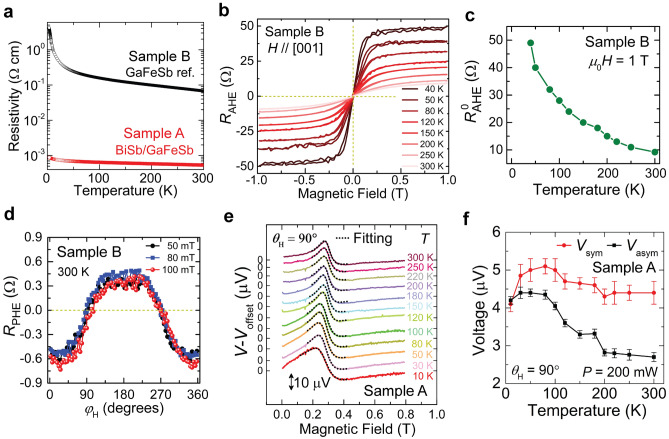
(**a**) Temperature dependence of the resistivity of the BiSb (7 nm)/(Ga,Fe)Sb (50 nm) heterostructure (sample A, red open circles) and the 50 nm-thick (Ga,Fe)Sb reference (sample B, black open circles). (**b**) Perpendicular magnetic field *μ*_0_***H*** dependence of the anomalous Hall resistance (*R*_AHE_) of sample B measured at various temperatures. (**c**) Saturation values (${\it\text{R}}_{{{\text{AHE}}}}^{{\text{0}}}$) of *R*_AHE_ as a function of temperature in sample B. (**d**) In-plane magnetic field direction (*φ*_H_) dependence of the planar Hall resistance (*R*_PHE_) of sample B with the applied magnetic field of 50 mT, 80 mT, and 100 mT measured at 300 K. *φ*_H_ is the angle of the in-plane magnetic field with respect to the [${1}\overline{\text{1}}{{0}}$] axis. (**e**) *μ*_0_***H*** dependences of the voltage signal *V* (offset voltage *V*_offset_ is subtracted) of sample A measured with 200 mW at various temperatures, ranging from 300 to 10 K, for *θ*_H_ = 90°. The black dotted curves represent fittings which were used to derive the magnitude of the symmetric voltage (*V*_sym_) and antisymmetric voltage (*V*_asym_) components. (**f**) *V*_sym_ and *V*_asym_ of sample A as a function of temperature. Red solid circles and black solid squares represent *V*_sym_ and *V*_asym_, respectively. The errors bars are standard deviations obtained from the fitting of the EMF peaks.

Next, we investigated the temperature dependence of the spin injection by spin pumping from (Ga,Fe)Sb to BiSb. Figure 4a shows the temperature dependence of the resistivity of samples A and B. We note that sample A (with the BiSb layer, red circles) shows larger conductivity than sample B (without BiSb, black circles) at all temperatures, which reflects the much higher electrical conductivity of the BiSb layer^37^. The estimated electrical conductivities (*σ*_GaFeSb_, *σ*_BiSb_) of (Ga,Fe)Sb and BiSb at 300 K are shown in Table 1. Because *σ*_BiSb_ (1.8 × 10^5^ Ω^−1^ m^−1^) >  > *σ*_GaFeSb_ (1.4 × 10^3^ Ω^−1^ m^−1^), the charge current mostly flows in the BiSb layer. In previous studies on BiSb thin films^35,45,46^, contribution of the topological surface states (SSs) to the total conductivity of the BiSb layer was estimated by using the parallel conduction model of the surface states and the bulk states. There, it was reported that a 10-nm-thick BiSb layer has a large bandgap of ~ 200 meV due to the quantum size effect, and that the SSs account for ~ 95% of the total current in BiSb even at room temperature. We used a similar parallel conduction model for our 7 nm-thick BiSb thin film and found that ~ 97% of the current flows in the SSs of BiSb at room temperature (see Sections 5–7 in S.I. for more details). This is also consistent with the recent observation using STM in 5.5 nm-thick BiSb thin films, where the band gap of BiSb is found to be further increased to 490 meV due to the strong quantum confinement, and that the Fermi level is in the band gap, so that most of the current flows in the SSs of BiSb^47^. We will use this information later for the estimation of the ISHA of BiSb.

**Table 1:** Parameters used for the ISHA estimation.

*l* (mm)	*d*_GaFeSb_ (nm)	*d*_BiSb_ (nm)	${\sigma}_{{\text{GaFe}}{\text{Sb}}}$ (Ω^−1^ m^−1^)	${\sigma}_{\text{BiSb}}$ (Ω^−1^ m^−1^)	*V*_ISHE_ (μV)
2	50	7	1.4 × 10^3^	1.8 × 10^5^	4.4 ± 0.3

*l* is the distance between the gold wire contacts, *d*_GaFeSb_ and *d*_BiSb_ are the thickness of the (Ga,Fe)Sb layer and the BiSb layer, respectively. *σ*_GaFeSb_ and *σ*_BiSb_ are the electrical conductivities of the (Ga,Fe)Sb layer and the BiSb layer estimated from transport measurements, respectively. *V*_ISHE_ (= *V*_sym_) is the ISHE voltage signal estimated by the maximum peak height of the symmetric component of the voltage signal after removing the offset value. All parameters are estimated at 300 K, at a microwave power of 200 mW with a magnetic field ***H*** direction of *θ*_H_ = 90° (***H*** // $[{1}\overline{\text{1}}{{0}}]$).

We then evaluated the temperature dependence of AHE in reference sample B [(Ga,Fe)Sb without BiSb]. For this purpose, we prepared a Hall bar of 200 μm (length) × 50 μm (width) and measured the temperature dependence of AHE with ***H*** // [001]. Figure 4b shows the anomalous Hall resistance (*R*_AHE_) as a function of temperature, which increases with decreasing temperature. We know that the saturation values of *R*_AHE_, ${{R}}_{\text{AHE}}^{0}$, reflects the AHE from the (Ga,Fe)Sb film; however, due to the presence of the ordinary Hall effect (OHE) along with the AHE, it is difficult to saturate *R*_AHE_, especially at high temperatures. Thus, we use *R*_AHE_ values at 1 T as ${{R}}_{\text{AHE}}^{0}$ which is closer to the saturation values of *R*_AHE_. Figure 4c shows ${{R}}_{\text{AHE}}^{0}$ as a function of temperature in sample B, where we see a clear increase in ${{R}}_{\text{AHE}}^{0}$ with decreasing temperature. We also measured the planar Hall resistance (*R*_PHE_) as a function of in-plane ***H*** angle *φ*_H_ (where *φ*_H_ is the angle between ***H*** and the [1$\overline{\text{1}}$0] axis), at a constant *μ*_0_*H* of 50 mT, 80 mT, and 100 mT at 300 K [Fig. 4d]. We note that in comparison with ${{R}}_{\text{AHE}}^{0}$ (= ~ 9 Ω), *R*_PHE_ (= ~ 0.6 Ω) is much smaller at 300 K. Furthermore, although *R*_PHE_ would follow cos(2*φ*_H_) dependence, *R*_PHE_ is actually dominated by the cos(*φ*_H_) component (see Fig. 4d). This cos(*φ*_H_) component is due to a small misalignment of the magnetic field from the xy plane, leading to the AHE contribution, while the PHE contribution of ${{R}}_{{\text{P}}{\text{HE}}}^{0}$ cos(2*φ*_H_) is negligible in Fig. 4d, where ${{R}}_{{\text{P}}{\text{HE}}}^{0}$ is the magnitude of *R*_PHE_. Therefore, we conclude ${{R}}_{{\text{P}}{\text{HE}}}^{0}$ << ${{R}}_{\text{AHE}}^{0}$ at room temperature. This result is consistent with the absence of the *V*_sym_ component in sample B observed in Fig. 3d.

Next, we measured the temperature dependence of the EMF at *θ*_H_ = 90° in sample A, as shown in Fig. 4e. We found that the EMF peak position *i.e.* resonance field (*μ*_0_*H*_R_) changes from 290 to 270 mT when temperature decreases from 300 to 10 K. This change is likely because *μ*_0_*H*_R_ depends on the magnetic anisotropy and the magnetization, both of which have temperature dependency. However, the magnitude and shape of the EMF signals depend on temperature. For example, the EMF peak at 10 K is significantly broader than that at 300 K. To understand this behavior, we fit Eq. (1) to the experimental data to estimate *V*_sym_ and *V*_asym_ at each temperature. Figure 4f shows the temperature dependence of *V*_sym_ and *V*_asym_. At low temperatures, we see clear enhancement of parasitic component *V*_asym_ by a factor of 3 at 10 K as compared with that at 300 K, which is consistent with the temperature dependence of *R*_AHE_ shown in the Fig. 4c. Meanwhile, *V*_sym_ shows close values (4.1 ± 0.2–5.1 ± 0.2 µV) at all temperatures, suggesting that the spin injection by spin pumping has small temperature dependence.

### Estimation of spin-to-charge conversion efficiency

In this section, we estimate the spin-to-charge conversion efficiency of BiSb, and discuss why BiSb is important to detect spin injection by spin pumping from (Ga,Fe)Sb. We first estimated the spin current density ${{j}}_{\text{S}}^{\text{BiSb/GaFeSb}}$ in sample A [BiSb/(Ga,Fe)Sb] by using the following equation^25^:

2 $${{j}}_{\text{S}}^{\text{BiSb/GaFeSb}} = \frac{{{g}}_{\text{r}}^{\uparrow \downarrow}{{\gamma}}^{2}{\left({\mu}_{0}{\it\text{h}}\right)}^{2}{\hbar}\left({4}{\pi}{\mu}_{0}{{{M}}}_{\text{S}}{\gamma} +\sqrt{{\left({4}{\pi}{\mu}_{0}{{{M}}}_{\text{S}}{\gamma}\right)}^{2} + { 4} {\omega}^{2}}\right)}{{8}{\pi}{\alpha}^{2}\left[{\left({{4}{\pi}{\mu}_{0}{{M}}}_{\text{S}}{\gamma}\right)}^{2} \, + 4 {\omega}^{2}\right]},$$

Here, *γ* = *gμ*_B_/$\hbar$ is the gyromagnetic ratio, where *g*, *μ*_B_, and $\hbar$ are the *g* factor, Bohr magneton, and Dirac’s constant, respectively, and *α* = $\sqrt{3}{\it\gamma}{\Delta H}_{\text{BiSb/GaFeSb}}/{2}{\it\omega}$ is the damping constant, and *μ*_0_Δ*H*_BiSb/GaFeSb_ (= 35 ± 0.5 mT) is the FMR spectral linewidth of sample A when ***H*** // [${1}\overline{\text{1}}{{0}}$]. Also, *μ*_0_*h*, *μ*_0_*M*_S_, and ${{g}}_{\text{r}}^{\uparrow \downarrow}$ are the microwave magnetic field, saturation magnetization, and real part of the spin mixing conductance, respectively. The real part of the spin mixing conductance is obtained by

3 $${{g}}_{\text{r}}^{\uparrow \downarrow} = \frac{{2}\sqrt{3}{\pi}{\mu}_{0}{{{M}}}_{\text{S}}{\gamma}{{d}}_{\text{GaFeSb}}}{{{g}}{\mu}_{\text{B}}{\omega}}\left({\Delta}{{H}}_{\text{BiSb/GaFeSb}}{-\Delta}{{H}}_{\text{GaFeSb}}\right),$$

where *μ*_0_Δ*H*_GaFeSb_ (= 32 ± 0.5 mT) is the FMR spectral linewidth of sample B when ***H*** // [${1}\overline{\text{1}}{{0}}$] and *d*_GaFeSb_ is the thickness of the (Ga,Fe)Sb film. Using Eqs. (2), (3), and the parameters shown in Table 2, we estimated the value of ${{g}}_{\text{r}}^{\uparrow \downarrow}$ and spin current density ${{j}}_{\text{S}}^{\text{BiSb/GaFeSb}}$ at the BiSb/(Ga,Fe)Sb interface at 300 K. The values of ${{g}}_{\text{r}}^{\uparrow \downarrow}$ and ${{j}}_{\text{S}}^{\text{BiSb/GaFeSb}}$ are estimated to be 1.20 × 10^19^ m^−2^ and (5.35 ± 0.21) × 10^–12^ J m^−2^, respectively.

**Table 2:** Parameters obtained by fitting our model to the experimental FMR spectra.

*μ*_0_Δ*H*_BiSb/GaFeSb_ (mT)	*μ*_0_Δ*H*_GaFeSb_ (mT)	*μ*_0_*h* (mT)	*μ*_0_*H*_R_ (mT)	*μ*_0_*M*_S_ (mT)	*γ* × 10^11^ (T^−1^ s^−1^)	*ω* × 10^10^ (s^−1^)	*g* factor
35 ± 0.5	32 ± 0.5	0.055	290	56 ± 2	1.84 ± 0.02	5.74	2.09 ± 0.03

Δ*H*_BiSb/GaFeSb_ and Δ*H*_GaFeSb_ are the FMR spectral linewidths for sample A and sample B when magnetic field ***H*** // $[{1}\overline{\text{1}}{{0}}]$, respectively; *μ*_0_*h, μ*_0_*H*_R_, *ω*, and *g* are the microwave magnetic field, resonance field, angular microwave frequency, and *g* factor, respectively. *γ* = *gμ*_B_/ℏ is the gyromagnetic ratio and *μ*_0_*M*_S_ is the saturation magnetization measured by SQUID.

Finally, we estimated ISHA by using the well-known expression^25^:

4 $${{V}}_{{\text{ISHE}} \, } =\frac{{{l}}{\theta}_{\text{ISHE}}{\lambda}_{\text{BiSb}}{\text{tanh}}\left({{d}}_{\text{BiSb}}/{2}{{\lambda}}_{\text{BiSb}}\right)}{{\sigma}_{\text{GaFeSb}}{{{d}}}_{\text{GaFeSb}} + {\sigma}_{\text{BiSb}}{{{d}}}_{\text{BiSb}}}\left(\frac{{2}{\text{e}}}{\hbar}\right){{j}}_{\text{S}}^{\text{BiSb/GaFeSb}},$$

where, *σ*_GaFeSb_, *σ*_BiSb_, *d*_GaFeSb_, *d*_BiSb_ are the electrical conductivities and thicknesses of the (Ga,Fe)Sb and BiSb layers, respectively; *l* is the length between the electrodes, *e* is the electron charge, $\hbar$ is the Dirac constant, and *θ*_ISHE_ is the inverse spin Hall angle. The estimated values of these parameters are shown in Table 1. As we discussed above, conduction of the charge current in BiSb occurs only in the SSs of the BiSb. Thereby, we modify Eq. (4) by replacing *d*_BiSb_ with *d*_S_ and *λ*_BiSb_ with *λ*_S_ in the numerator part, where *d*_S_ and *λ*_S_ are the thickness and spin diffusion length of the surface states in the BiSb layer. It is reported that the surface state thickness and spin diffusion length of Bi is *d*_S_ = 2.0 ± 0.2 nm and *λ*_S_ = 2.4 ± 0.3 nm^54^. Because BiSb in this work is a topological insulator with a band gap significantly larger than that of Bi, the surface state thickness is expected to be thinner than that of Bi. Therefore, we assume that *d*_S_ ~ *λ*_S_ = 1.0–1.2 nm in Bi_0.85_Sb_0.15_^41^. The modified expression for ISHA estimation based on the dominant surface conduction is given by

5 $${{V}}_{\text{ ISHE}} = \frac{{{l}}{\theta}_{\text{ISHE}}{{{d}}}_{\text{S}}{\text{tanh}}\left({1}/{2}\right)}{{\sigma}_{\text{BiSb}}{{{d}}}_{\text{BiSb}}}\left(\frac{{2}{\text{e}}}{\hbar}\right){{j}}_{\text{S}}^{\text{BiSb/GaFeSb}},$$

Using the parameters in Table 1 and Eq. (5), we estimated ISHA ${\theta}_{\text{ISHE}}$ ~ 2.1–2.6. This ${\theta}_{\text{ISHE}}$ is close to the SHA value *θ*_SHE_ ~ 3.2 reported for non-epitaxial BiSb grown by MBE^35^ and 2.0–2.4 for sputtered BiSb^36,37^. As shown in Table 3, the spin-to-charge conversion efficiency at 300 K of BiSb is much larger than those reported in literature. Nevertheless, in the present study, we deposited poly-crystalline BiSb by sputtering on the etched surface of MBE-grown (Ga,Fe)Sb, which may have led to lower ISHA. Thus, an even higher ISHA value may be obtained by growing both BiSb and (Ga,Fe)Sb epitaxially. This large spin-to-charge conversion efficiency of BiSb is the key to detect spin injection by spin-pumping from (Ga,Fe)Sb with the tiny *M*_S_ ~ 45 emu/cc at room temperature, which is 40 times smaller than that of Fe (1800 emu/cc).

**Table 3:** Comparison of the spin-to-charge conversion efficiency between our sample with other heterostructures.

Sample structure	*T* (K)	${\theta}_{\text{ISHE}}$ at 300 K	Reference
Ni_81_Fe_19_/*p*-Si	300	0.0001	Ref.^55^
Co/Pt and Co/Cu/Pt	300	0.056	Ref.^56^
Au/Fe/Ag/*α*-Sn	300	0.62	Ref.^57^
SiO_2_/NiFe/Bi_2_Se_3_	15–300	0.0093	Ref.^58^
Ni_81_Fe_19_/Sn-Bi_2_Te_2_Se	15–300	0.0001	Ref.^53^
(Ga,Fe)Sb/BiSb	300	2.1–2.6	This work

*T* is a temperature at which ISHE voltage signals were measured and *θ*_ISHE _is the spin-to-charge conversion efficiency.

## Conclusion

We have demonstrated spin injection by spin pumping and detection by ISHE in a BiSb/(Ga,Fe)Sb heterostructure at room temperature. This work is the first spin injection experiment using high-*T*_C_ FMS. From the temperature, microwave power, and magnetic field direction dependences of the ISHE voltage as well as the spin pumping experiment on the reference (Ga,Fe)Sb sample, we conclude that the symmetric voltage component obtained in the BiSb/(Ga,Fe)Sb sample has a negligible galvanomagnetic effect. The result presented in this study opens new opportunities for semiconductor-spintronics device applications operating at room temperature.

## Methods

### Sample growth

The growth of the sample structure is divided into two parts. We first grew a heterostructure composed of (Ga_0.75_,Fe_0.25_)Sb (50 nm)/AlSb (100 nm)/AlAs (10 nm)/GaAs (100 nm) on a semi-insulating (SI) GaAs (001) substrate with a growth rate of 0.5 μm/h by molecular beam epitaxy (MBE), followed by growth of a 2 nm-thick GaSb cap layer to avoid surface oxidation. The substrate temperature (*T*_S_) was 550 °C for the GaAs and AlAs layers, 470 °C for the AlSb layer, 250 °C for the (Ga,Fe)Sb and GaSb layer. After that, we preserved a half part of the sample as a reference sample and transferred the other half part of the sample to the sputtering system for the BiSb deposition. There, we first etched the GaSb cap layer using Ar-plasma at room temperature. Then, we deposited a 7 nm-thick Bi_0.85_Sb_0.15_ layer with a rate of 5.8 nm/min on top of the 50 nm-thick (Ga,Fe)Sb layer by co-sputtering Bi and Sb targets with Ar-plasma at room temperature. During the MBE growth, the crystallinity and surface morphology of the samples were monitored in situ by reflection high-energy electron diffraction (RHEED) along the [$\overline{\text{1}}{{10}}$] azimuth with respect to the GaAs (001) substrate.

### Characterizations

The MCD measurements were performed using a J700 system (built by JASCO Corporation) equipped with an electromagnet and a cryostat. AHE measurements were performed on samples etched into 50 × 200 μm Hall bars using photolithography and ion milling.

### Spin pumping measurement

In spin pumping experiments, we used a JEOL electron spin resonance (ESR) spectrometer whose cavity resonates in the transverse electric (TE_011_) mode with a microwave frequency *f* of 9.14 GHz (*X*-band). We cut the samples into a 3 × 1 mm^2^ piece with edges along the ${[110]}$ (3 mm) and ${[1}\overline{\text{1}}{0]} \,$ (1 mm) axes. For electrical measurements, we have connected the two gold wires to the indium contacts at both edges of the sample with a distance of *l* = 2 mm. The sample was placed on the center of a quartz rod and inserted in the center of the microwave cavity. For the measurements, a static magnetic field *μ*_0_***H*** is applied along the [1$\overline{\text{1}}$0] axis in the film plane (except for the measurements with varying *θ*_H_), which corresponds to the easy magnetization axis of (Ga,Fe)Sb. A microwave magnetic field *μ*_0_***h*** is applied along the [110] axis. Also, an ac modulation field *μ*_0_***H***_ac_ (1 mT, 100 kHz) parallel to ***H*** is superimposed to obtain the FMR spectrum in its derivative form. The voltage between the indium contacts is detected by a nano-voltmeter (see S.I. Section 4).

## Supplementary Information

Supplementary Information.

## Data Availability

The data that support the findings of this study are available from the corresponding author upon reasonable request.
