# Supplementary material for: Room-temperature spin injection from a ferromagnetic semiconductor
Source: Sci Rep. 2023 Feb 7;13:2181. doi: 10.1038/s41598-023-29169-9 (PMC9905071; doi:10.1038/s41598-023-29169-9)
Supplement: Supplementary file 1 — Supplementary Information. [file 41598_2023_29169_MOESM1_ESM.pdf]

Supplementary Information

Room-temperature spin injection from a ferromagnetic semiconductor

Shobhit Goel,[1,2] Nguyen Huynh Duy Khang,[3,4] Yuki Osada,[3] Le Duc Anh,[1,5,6,7]
Pham Nam Hai,[2,3,7] and Masaaki Tanaka[1,2,7,8]

[1]*Department of Electrical Engineering and Information Systems, The University of Tokyo, 7-3-1 Hongo, Bunkyo-ku, Tokyo 113-8656, Japan.*

[2]*CREST, Japan Science and Technology Agency, 4-1-8 Honcho, Kawaguchi, Saitama 332-0012, Japan.*

[3]*Department of Electrical and Electronic Engineering, Tokyo Institute of Technology, 2-12-1 Ookayama, Meguro-ku, Tokyo 152-8550, Japan.*

[4]*Department of Physics, Ho Chi Minh City University of Education, 280 An Duong Vuong Street, District 5, Ho Chi Minh City 738242, Vietnam.*

[5]*Institute of Engineering Innovation, The University of Tokyo, 7-3-1 Hongo, Bunkyo-ku, Tokyo 113-8656, Japan.*

[6]*PRESTO, Japan Science and Technology Agency, 4-1-8 Honcho, Kawaguchi, Saitama 332-0012, Japan*

[7]*Center for Spintronics Research Network (CSRN), The University of Tokyo, 7-3-1 Hongo, Bunkyo, Tokyo 113-8656, Japan.*

[8]*Institute for Nano Quantum Information Electronics, The University of Tokyo, 4-6-1 Komaba, Meguro-ku, Tokyo 153-8505, Japan.*

1. Magnetic circular dichroism (MCD) measurements

We characterized the intrinsic ferromagnetism of (Ga,Fe)Sb before and after depositing BiSb by sputtering, using the magnetic circular dichroism (MCD) spectroscopy in a reflection setup. The reflection MCD intensity is given by the difference in the optical reflectivity for right ($R_{\sigma+}$) and left ($R_{\sigma-}$) circular polarization of light. The MCD intensity is expressed as

$MCD = \frac{90}{\pi} \frac{(R_{\sigma+} - R_{\sigma-})}{2R} = \frac{90}{\pi} \frac{1}{2R} \frac{dR}{dE} \Delta E$, where R is the optical reflectivity, E is the photon energy and ΔE is the Zeeman splitting energy. Since MCD is proportional to dR/dE and ΔE, it directly probes the spin-polarized band structure of the measured material. Thus, MCD is a powerful tool to characterize the intrinsic magnetic properties of (Ga,Fe)Sb. Figures S1 (a) and (b) show the MCD spectra of sample A [BiSb (7 nm) / (Ga,Fe)Sb (50 nm)] and sample B [(Ga,Fe)Sb (50 nm)], respectively, measured at 5 K with a perpendicular magnetic field of 0.2 T, 0.5 T, and 1 T, normalized by the intensity of the E_1 peak at 1 T. As shown in Figs. S1(a) and (b), the MCD spectra have the E_1 peak around ~ 2.4 eV, reflecting the band structure of zinc-blende (Ga,Fe)Sb in both samples. In Figs. S1(a) and (b), normalized MCD spectra which were measured at various magnetic fields are overlapped on one spectrum, indicating that the ferromagnetism in (Ga,Fe)Sb comes from a single phase, that is the zinc-blende (Ga,Fe)Sb. Also, Figs. S1(c) and (d) show the magnetic-field ($\mu_0 H$) dependence of the MCD intensity at E_1 of both the samples, which show clear hysteresis even at room temperature. Thus, the ferromagnetism and the related magnetic properties are maintained in the (Ga,Fe)Sb before and after depositing BiSb without destroying the intrinsic ferromagnetism of zinc-blende (Ga,Fe)Sb. We note that the coercive force of the BiSb/(Ga,Fe)Sb (sample A) is smaller than that of the (Ga,Fe)Sb (sample B) at 5 K, probably due to over-etching of the cap layer before depositing the BiSb layer. The coercive force is caused by domain wall pinning at crystal defects, such as defects at the cap layer/(Ga,Fe)Sb interface. By slightly over-etching the cap layer, such defects may be removed and the coercive force of (Ga,Fe)Sb in sample A became smaller.

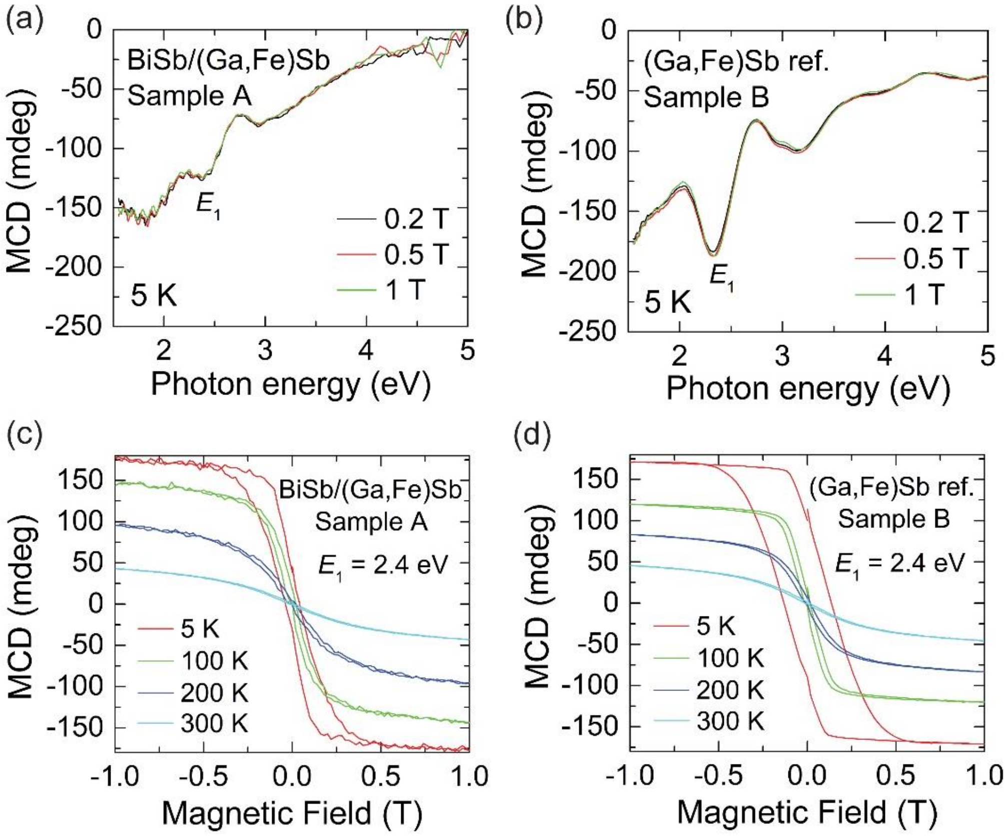

Figure S1. (a) and (b) MCD spectra of the (a) BiSb (7nm) / (Ga,Fe)Sb (50 nm) heterostructure (sample A) and (b) (Ga,Fe)Sb (50 nm) reference (sample B) at 5 K with a magnetic field of 0.2 T, 0.5 T, and 1 T applied perpendicular to the film plane. The MCD spectra measured at 0.2 T and 0.5 T are normalized to that at 1 T by the intensity of the E_1 peak. (c) and (d) $MCD - \mu_0 H$ curves at E_1 of (c) BiSb/(Ga,Fe)Sb (sample A) and (d) (Ga,Fe)Sb reference (sample B) at various temperatures.

2. Superconducting quantum interference device (SQUID) magnetometry measurements

Figures S2(a) and (b) show the magnetic-field dependence of the magnetization ($M - \mu_0 H$ characteristics) of sample A [BiSb/(Ga,Fe)Sb] and sample B [(Ga,Fe)Sb ref.] measured at 300 K, with a magnetic field $\mu_0 \boldsymbol{H}$ applied along the in-plane [110] axis (red solid circles). In both samples, the saturation magnetization is nearly the same [$(4.50 \pm 0.1) \times 10^4$ A/m],

indicating that the ferromagnetism of (Ga,Fe)Sb did not change before and after depositing BiSb. From Fig. S2, the estimated saturation magnetization $\mu_0 M_S$ is 0.056 ± 0.002 T. We used this $\mu_0 M_S$ value for the estimation of spin current and magnetic anisotropy.

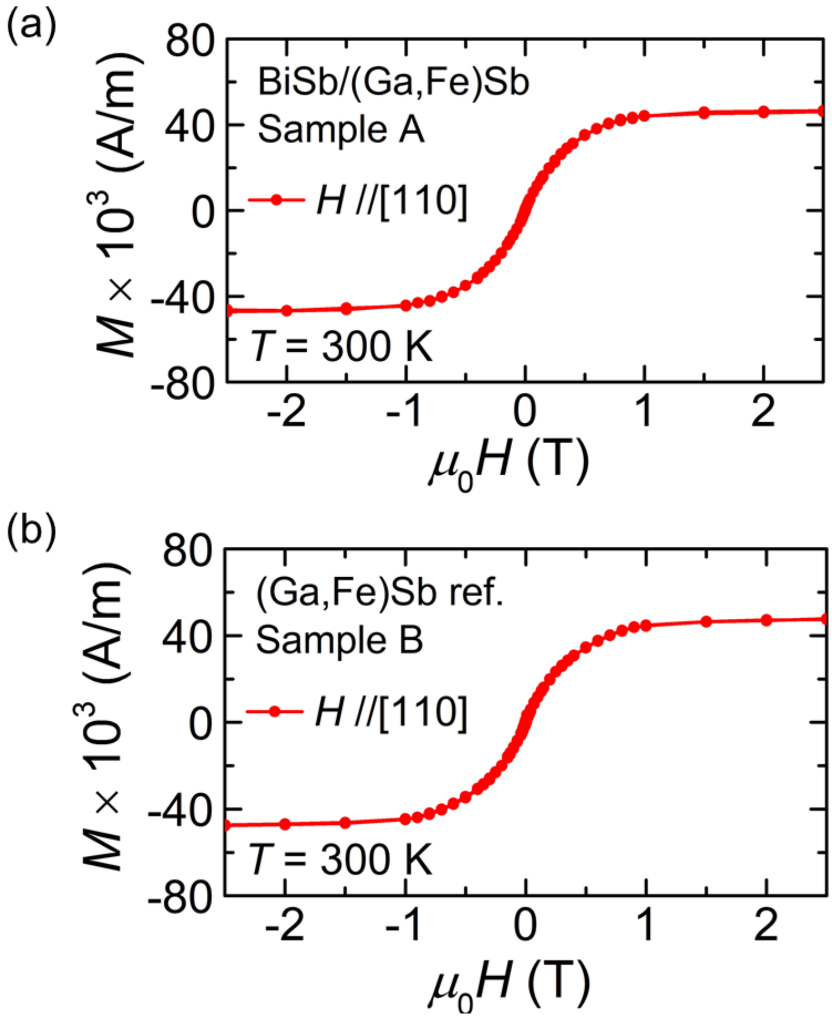

Figure S2. (a) and (b) Magnetization hysteresis curves ($M - \mu_0 H$) measured at 300 K for the (a) BiSb/(Ga,Fe)Sb (sample A) and (b) reference (Ga,Fe)Sb (sample B), when the magnetic field $\mu_0 \boldsymbol{H}$ is applied in the film plane along the [110] axis.

3. Magnetic field direction (θ_H) dependence of ferromagnetic resonance

Figures S3(a) and (b) show the FMR spectra for sample A [(Ga,Fe)Sb with BiSb] and sample B [(Ga,Fe)Sb without BiSb], respectively, measured at various magnetic field directions (θ_H) at 300 K. In both samples, the resonance field $\mu_0 H_R$ of the FMR spectra changed from 290 mT to 336 mT when θ_H is changed from ±90° ($\boldsymbol{H}$ // $[1\bar{1}0]$ and $[\bar{1}10]$) to 0° ($\boldsymbol{H}$ // [001]), respectively. As shown in Fig. S3, the FMR spectra and peak positions are almost the same before and after depositing BiSb, thus the magnetic anisotropy remains the same in sample A and sample B. This indicates that the ferromagnetic properties of (Ga,Fe)Sb are not affected by etching or BiSb overgrowth.

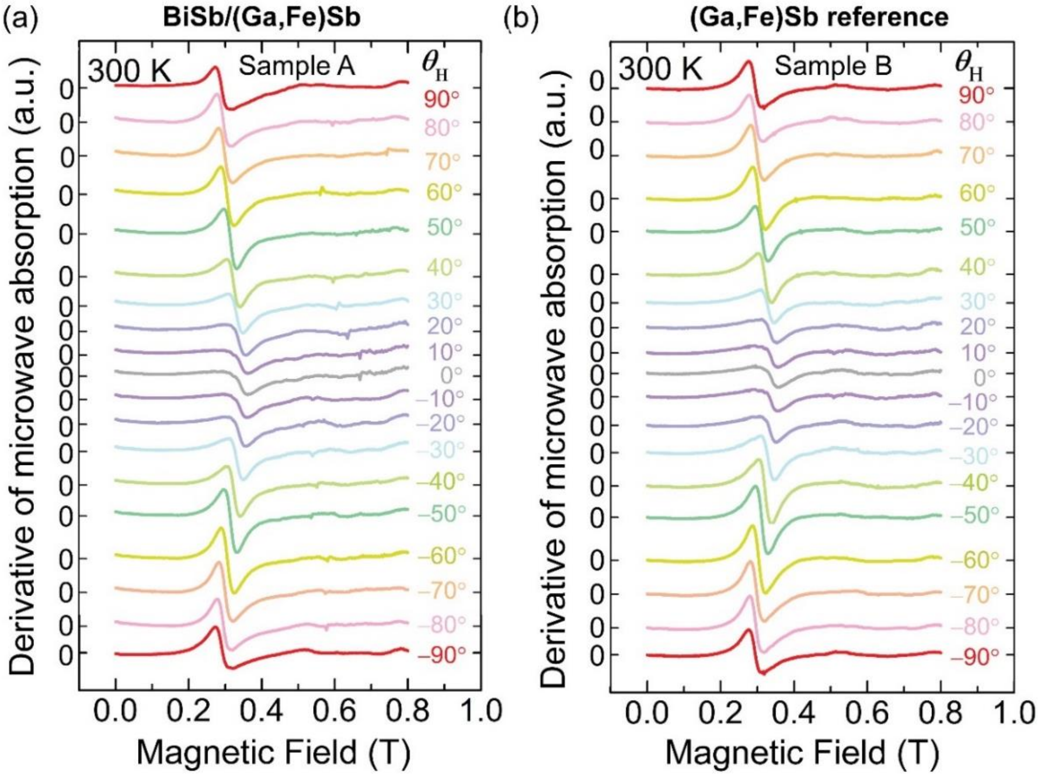

Figure S3. Ferromagnetic resonance (FMR) spectra at 300 K of (a) sample A [BiSb/(Ga,Fe)Sb], and (b) sample B [(Ga,Fe)Sb without BiSb], at various magnetic field directions (θ_H). The used microwave power is 200 mW. θ_H is defined in Fig. S4(a).

Next, we have used the fitting equations which were derived from the Smith-Beljers relation[S1] and the Landau-Lifshitz Gilbert (LLG) equation, to fit them to the experimental angular dependence of FMR (Please see our previous paper ref S2 for the detailed method of how to obtain fitting equations). The fitting equation for the rotation of ***H*** in the perpendicular plane ($\varphi_H = \varphi_M = -45°$), *i.e.*, in the (110) plane and in the film plane ($\theta_H = \theta_M = 90°$) *i.e.*, in the (001) plane, are expressed as Eq. (S1) and Eq. (S2), respectively.

$$\left(\frac{\omega}{\gamma}\right)^2 = \left[\mu_0 H_R \cos(\theta_H - \theta_M) + \left(-\mu_0 M_S + \mu_0 H_{2\perp} - \mu_0 H_{2//}\right)\cos^2\theta_M + \mu_0 H_{2//}\right] \times \left[\mu_0 H_R \cos(\theta_H - \theta_M) + \left(-\mu_0 M_S + \mu_0 H_{2\perp} - \mu_0 H_{2//}\right)\cos 2\theta_M\right] \quad \text{(S1)}$$

$$\left(\frac{\omega}{\gamma}\right)^2 = \left[\mu_0 H_R \cos(\varphi_H - \varphi_M) + \mu_0 M_S - \mu_0 H_{2\perp} + \mu_0 H_{2//}\sin^2\left(\varphi_M - \frac{\pi}{4}\right)\right] \times \left[\mu_0 H_R \cos(\varphi_H - \varphi_M) - \mu_0 H_{2//}\cos\left(2\varphi_M - \frac{\pi}{2}\right)\right] \quad \text{(S2)}$$

Here, μ_0 is the vacuum permeability, $\omega = 2\pi f$ is the angular frequency of magnetization precession where f is the microwave frequency, $\gamma = g\mu_B/\hbar$ is the gyromagnetic ratio where g is the Landé g-factor, μ_B is the Bohr magneton, and $\hbar$ is the reduced Planck constant. $\mu_0 H_R$, $\mu_0 H_{2\perp}$, $\mu_0 H_{2//}$, and $\mu_0 M_S$ are the resonance field, perpendicular uniaxial magnetic anisotropy, in-plane uniaxial magnetic anisotropy, and saturation magnetization, respectively. θ_H and θ_M are the out-of-plane magnetic field angle and magnetization angle, respectively. Here, θ_H and θ_M are defined with respect to the [001] axis [Fig. S4 (a)]. φ_H and φ_M are the in-plane magnetic field angle and magnetization angle, respectively. Here, φ_H and φ_M are defined with respect to the [100] axis [Fig. S4 (b)]. θ_M and φ_M are given by:

$$\left(-\mu_0 M_S + \mu_0 H_{2\perp}\right)\sin 2\theta_M = 2\mu_0 H_R \sin(\theta_M - \theta_H) \quad \text{(S3)}$$

$$\mu_0 H_{2//}\sin\left(2\varphi_M - \frac{\pi}{2}\right) = 2\mu_0 H_R \sin(\varphi_M - \varphi_H) \quad \text{(S4)}$$

Using $\mu_0 M_S$ (= 0.056 ± 0.002 T), which was obtained from the SQUID result, together with $\mu_0 H_{2\perp}$, $\mu_0 H_{2//}$ and γ values, we reproduced the experimental angular dependence of FMR fields very well, as shown in Fig. S4 (c) and (d). The estimated values of $\mu_0 H_{2\perp}$ and $\mu_0 H_{2//}$ are 0.026 ± 0.002 T and 0.004 ± 0.002 T, respectively; and $\gamma (= g\mu_B/\hbar)$ after substituting g = 2.09 ± 0.03 is (1.84 ± 0.02) × 10^{11} (T^{-1} s^{-1}).

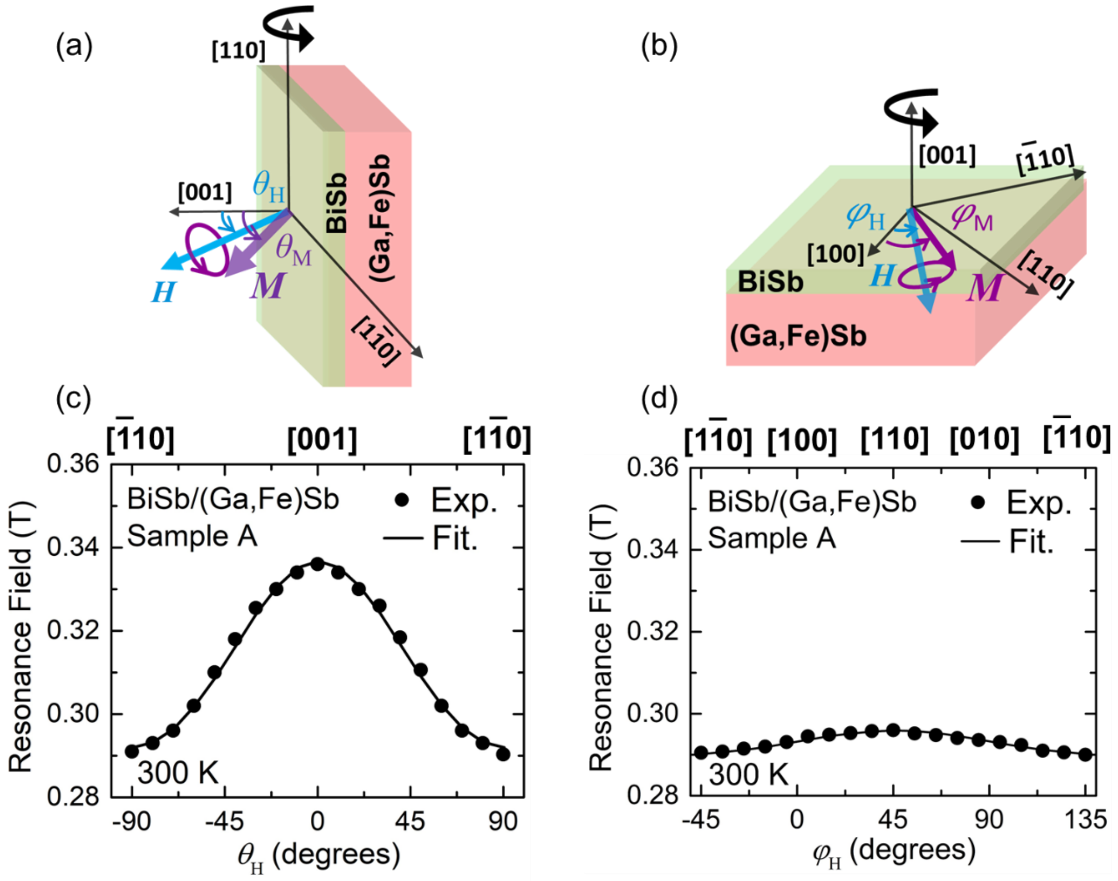

Figure S4. Coordinate system used in our FMR measurement. (a) Sample is rotated in the perpendicular plane [(110) plane]. θ_H and θ_M are the magnetic field and magnetization angles with respect to the perpendicular [001] axis, respectively. (b) Sample is rotated in the film plane [(001) plane]. φ_H and φ_M are the magnetic field and magnetization angles with respect to the in-plane [100] axis, respectively. (c) Resonance field ($\mu_0 H_R$) (solid symbols) as a function of the magnetic field angle θ_H at 300 K. The solid line is a fitting curve using Eq. (S1). (d) Resonance field ($\mu_0 H_R$) (solid symbols) as a function of the magnetic field angle φ_H at 300 K. The solid line is a fitting curve using Eq. (S2).

4. Spin pumping measurement

In spin pumping experiments, we used a JEOL electron spin resonance (ESR) spectrometer whose cavity resonates in the transverse electric (TE_{011}) mode with a microwave frequency f of 9.14 GHz (X-band). We cut the samples into a 3 × 1 mm^2 piece with edges along the $[110]$ (3 mm) and $[1\bar{1}0]$ (1 mm) axes. For electrical measurements, we have connected the two gold wires to the indium contacts at both edges of the sample with a distance of l = 2 mm apart as shown in Fig. S5 (same as Fig. 1(e) in the main manuscript). The sample was placed on the center of a quartz rod and inserted in the center of the microwave cavity. For the measurements, a static magnetic field $\mu_0\boldsymbol{H}$ is applied along the $[1\bar{1}0]$ axis in the film plane (except for the measurements with varying θ_H), which corresponds to the easy magnetization axis of (Ga,Fe)Sb. A microwave magnetic field $\mu_0\boldsymbol{h}$ is applied along the [110] axis [Fig. S5 and Fig. 1(e) in main manuscript]. Also, an ac modulation field $\mu_0\boldsymbol{H}_{ac}$ (1 mT, 100 kHz) parallel to $\mu_0\boldsymbol{H}$ is superimposed to obtain the FMR spectrum in its derivative form. The voltage between the indium contacts is detected by a nano-voltmeter. We have measured the FMR signal and the voltage signal V between the indium contacts simultaneously by sweeping the magnitude of $\boldsymbol{H}$. When the FMR condition is satisfied, a pure spin current with the spin polarization parallel to the magnetization precession axis in the (Ga,Fe)Sb layer is injected into the BiSb layer by spin pumping. This injected spin current is converted to a charge current by ISHE, which generates a voltage signal between the edges in the BiSb layer. As discussed in the main manuscript, this voltage signal sometimes contains galvanomagnetic effects, which is originated due to the shift in the sample alignment from the center position inside the cavity. As a result, the microwave electric field produces galvanomagnetic effects. These galvanomagnetic effects can be separated from the inverse spin Hall effect (ISHE) signal by

decomposing the voltage signals into symmetric and asymmetric components using Eq. (1) in the main manuscript. In the case of the ideal microwave cavity, *i.e*. the 90° phase difference between the radio frequency (rf) electric field and the rf magnetic field, the symmetric component consists of the planar Hall effect (PHE) and ISHE, while the asymmetric component composed of only the anomalous Hall effect (AHE). However, the situation becomes complicated if the phase difference is shifted from 90°. Thus, to avoid such complications, we assumed an ideal microwave cavity in this work.

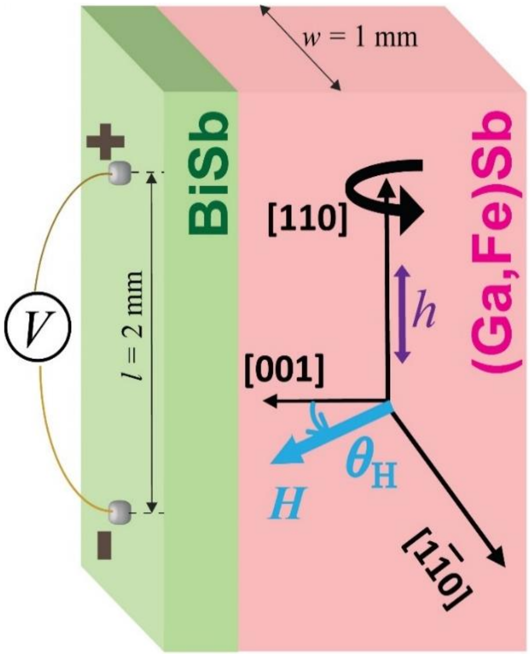

Figure S5. Sample alignment and coordinate system used in the spin pumping measurement. A microwave magnetic field $\mu_0\boldsymbol{h}$ was applied along the [110] axis of the sample. θ_H is the angle of the magnetic field $\boldsymbol{H}$ with respect to the [001] axis, respectively, where $\boldsymbol{H}$ is in the (110) plane. l is the distance between the two gold wires connected to the indium contacts.

Next in order to detect the spin current from the (Ga,Fe)Sb layer using spin pumping, we first tried to use heavy metal such as Pt with spin Hall angle (SHA) = 0.056 [S3], as a spin detector of (Ga,Fe)Sb. We grew a 10 nm-thick Pt layer by sputtering over a 50 nm-thick

(Ga,Fe)Sb film grown by molecular beam epitaxy. Figure S6 shows FMR (top panel) and EMF (bottom panel) signals of Pt/(Ga,Fe)Sb measured at 200mW and at 300 K. Even though we observed clear FMR spectra, we did not see a voltage peak at the resonance field ($\mu_0 H_R$) in the EMF signal. This is because the magnetization of (Ga,Fe)Sb is only 45 emu/cc (much smaller than 1800 emu/cc of Fe), and the ISHE voltage is proportional to the magnetization of the ferromagnetic layer; thus it would be impossible to detect the ISHE voltage without a material of a large spin-to-charge conversion efficiency *i.e.* large SHA (as discussed in the main manuscript). This indicates the importance of choosing sputtered BiSb with SHA = 10.7 [S4] as a spin detector for (Ga,Fe)Sb.

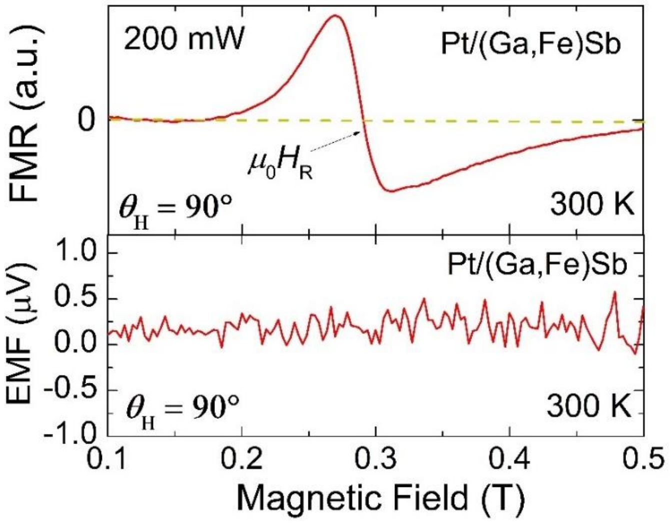

Figure S6. FMR (top panel) and EMF (bottom panel) signals of Pt (10 nm) /(Ga,Fe)Sb (50 nm) measured at 300 K and θ_H = 90°

5. Bi_xSb_{1-x} thin films with metallic surface states and insulting bulk states

To characterize the properties of $Bi_{1-x}Sb_x$, we have grown $Bi_{1-x}Sb_x$ thin films on semi-insulating GaAs(111)A substrates by molecular beam epitaxy (MBE). The growth procedure is as follows: After desorption of the surface oxide layer of the GaAs(111)A substrates by thermal annealing, we first grew a GaAs buffer layer at a substrate temperature of 570 °C to obtain

atomically smooth surface. Then, we grew a $Bi_{1-x}Sb_x$ thin film at 100 – 250 °C, depending on the Sb concentration x ranging from 0% to 32%. Figures S7 (a) − (d) show the temperature dependence of the electrical resistivity of various $Bi_{1-x}Sb_x$ thin films in the bulk topological insulator (TI) region (7% ≤ x ≤ 22%), while Figures S7 (e) − (f) show those of $Bi_{1-x}Sb_x$ thin films with high Sb concentrations, slightly outside of the bulk TI region (22% < x). We observed insulating behavior for thick films and metallic behavior for thin films. These features are similar to those observed for Bi thin films[S5], in which it was proven that Bi thin films have metallic surface states. Meanwhile, there is a strong quantum confinement effect in Bi thin films, which significantly increases the band gap when the thickness becomes smaller. At a small film thickness, because the Fermi level lies in the band gap, the bulk conduction due to intrinsic electron/hole carriers is significantly suppressed, thus the surface conduction is dominant.

Our experimental results shown in Fig. S7 [published in ref. S6] reproduce those of ref. S5. Note the metallic behavior in the 10 nm-thick $Bi_{0.93}Sb_{0.07}$ (see Fig. S7(a)), and 10 nm-thick $Bi_{0.89}Sb_{0.11}$ (see Fig. S7(c)) thin films. These results indicate that surface-state transport becomes dominant in BiSb when the film thickness is less than 10 nm. In section 6, we show how we estimate the contribution of the surface-state transport.

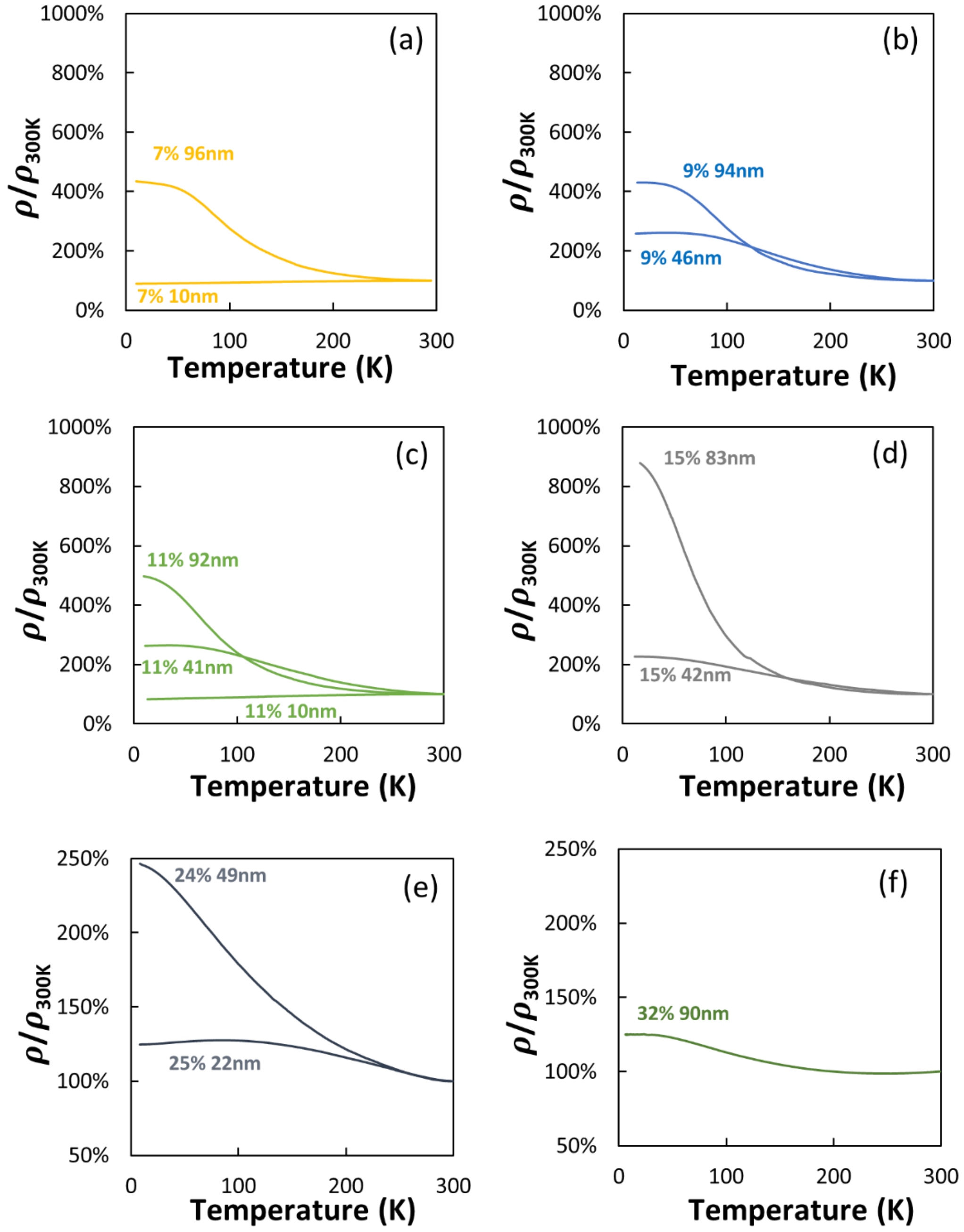

Figure S7. Temperature dependence of the resistivity of various $Bi_{1-x}Sb_x$ thin films. (a) x = 7%, (b) x = 9%, (c) x = 11%, (d) x = 15%, (e) x = 24 – 25%, (f) x = 32%, demonstrating metallic surface states and insulating bulk states, inside and slightly outside of the bulk TI regions. Data from Ref. S6.

6. Estimation of surface-state contribution

We estimate the band gap E_g of $Bi_{1-x}Sb_x$ thin films from the temperature dependence of the resistivity, using the surface and bulk parallel conduction model[S5]. Here, the total conductance $G(T) = G_S + G_0 \exp(-\frac{E_g}{2k_B T})$ includes the semiconducting bulk conductance $G_0 \exp(-\frac{E_g}{2k_B T})$ and the metallic surface state conductance G_S which is temperature-independent. Note that the thermal activation term of the bulk conductance reflects the fact that the Fermi level is in the band gap. Representative fitting was shown in the Fig. S8(a). The band gap data for samples with a film thickness t ~ 90 nm at various Sb compositions are shown in Fig. S8(b). The band gap of $Bi_{1-x}Sb_x$ increases with x and reaches the maximum at x ~ 15%, then decreases. Our transport results shown in Fig. S7 are well explained by the band structure of $Bi_{1-x}Sb_x$. The band gap values of $Bi_{1-x}Sb_x$ thin films are larger than those of bulk $Bi_{1-x}Sb_x$ (black line) due to the quantum size effect even at the film thickness of 90 nm. Furthermore, the band gap exists not only inside the bulk TI regions ($0.07 \leq x \leq 0.22$) but also outside of the bulk TI region ($x < 0.07$ and $0.22 < x$), which is also caused by the quantum-size effect.

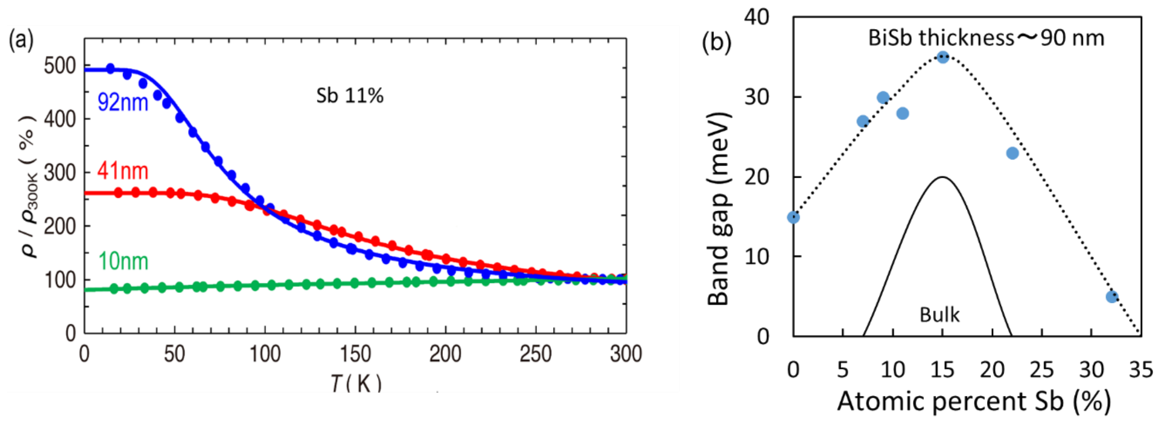

Figure S8. (a) Representative experimental data (dots) and fitting (lines) by a parallel

conduction model of BiSb, for the temperature dependence of the resistivity in $Bi_{0.89}Sb_{0.11}$ thin films with various thicknesses; t = 10 nm (green), 41 nm (red), and 92 nm (blue). (b) Band gap of $Bi_{1-x}Sb_x$ thin films with t = ~ 90 nm as a function of Sb concentration. The black line shows the bulk band gap. Data from Ref. S6.

We observed similar behavior for sputtered BiSb thin films, and confirmed that the surface conduction is dominant at $t \leq$ 10 nm [S7]. Figure S9 shows the conductivity σ_{BiSb} of sputtered BiSb thin films, grown on a Sapphire substrate, as a function of the BiSb thickness t from 28 nm to 4 nm measured at 300 K. For 28 nm ≥ t ≥ 7 nm, the conductivity keeps increasing as t decreases, consistent with the dominant surface conduction. Below 7 nm (t < 7 nm), the conductivity abruptly decreases; we believe that this is due to the quantum interference of the top and bottom surface. This quantum interference leads to band gap opening of the surface states [S8]. The surface states become less metallic and slightly semiconducting with a small band gap, thus the conductivity decreases. Because the band gap opening is not suitable for our purpose of measuring the inverse spin Hall effect coming from the topological surface states, we have chosen the thickness of 7 nm, where the surface conduction is maximum and thus the conductivity is maximum.

Next, the surface conductance G_s can be obtained by $G_S \sim G^{exp}(T \to 0\,\mathrm{K})$. The contribution of the surface states to the total conductivity at room temperature is then given by $\Gamma = \frac{G_S}{G}$ ~ 97% for the 7 nm-thick $Bi_{0.85}Sb_{0.15}$ thin film (estimated in Sec 7).

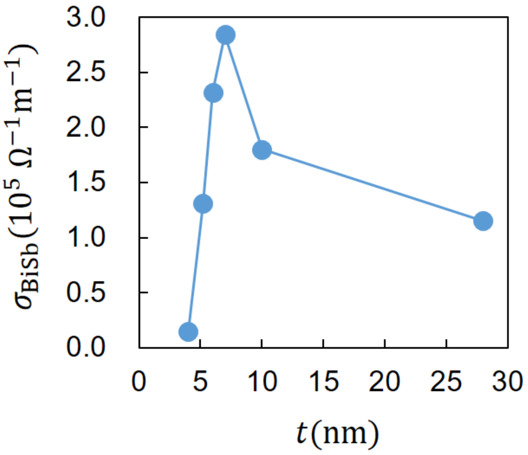

Figure S9. Thickness (t) dependence of the conductivity of BiSb (σ_{BiSb}) measured at 300 K

7. Surface state conduction in the 7-nm-thick BiSb layer (this work)

To show that the surface conductance is dominant in our BiSb layer, we demonstrate the same conductance of two layers of BiSb grown on the same substrate [semi-insulating (SI) GaAs (001) substrates] in two different samples. By fitting our model described in Sec 6, to the measured transport data of the BiSb layers of different samples, we show that surface state conduction is dominant in our 7-nm-thick BiSb layer.

(a) Preparation of BiSb layers grown on different buffer layers on GaAs (001) semi-insulating (SI) substrates.

We use the first sample: sample A [BiSb/(Ga,Fe)Sb], where a 7-nm-thick BiSb layer was grown on top of the 50-nm-thick (Ga,Fe)Sb layer after etching the 2-nm-thick GaSb cap layer in the reference (Ga,Fe)Sb sample [sample B, Fig. S10(a)] (Please see the main manuscript for detailed growth procedure). Because sample A [Fig. S10(b)] has several buffer layers (GaFeSb/AlSb/AlAs/GaAs), which may have parallel conduction and may contribute to the total conductivity (*whose possibility is negligible because reference sample B in the main manuscript shows the conductivity of ~ 10^3 $\Omega^{-1}m^{-1}$, much lower than the conductivity of BiSb ~*

10^5 $\Omega^{-1}m^{-1}$). To check this possibility, we prepared a second sample by growing a 7-nm-thick BiSb layer directly (*without any buffer layers*) on a SI GaAs (001) substrate [sample C, Fig. S10(c)] after etching the oxidized GaAs surface, using the same growth condition and procedure as the sample A [BiSb/(Ga,Fe)Sb]. Figures S10(d)–(g) show a comparison of reflection high-energy electron diffraction (RHEED) patterns during the growth of sample A [BiSb/(Ga,Fe)Sb] and sample C [BiSb/GaAs]. As described in the main manuscript, in both sample A and sample C, after etching the top surface (GaSb cap layer in sample A and GaAs oxidized surface in sample C), we observed spotty RHEED patterns [Figs. S10(d) and (e)], indicating that the (Ga,Fe)Sb surface in sample A preparation and the GaAs surface in sample C preparation are exposed, respectively. Then, we deposited BiSb and ring RHEED patterns appeared, indicating that poly-crystalline BiSb layers are formed [Figs. S10(f) and (g)]. Thus, we confirmed the successful growth of poly-crystalline BiSb in both samples. Note that achieving poly-crystalline RHEED of BiSb in sample C is not only important to have the same quality of BiSb in both samples (sample A and sample C), but also important to compare the intrinsic surface conductance in the BiSb layer in both samples.

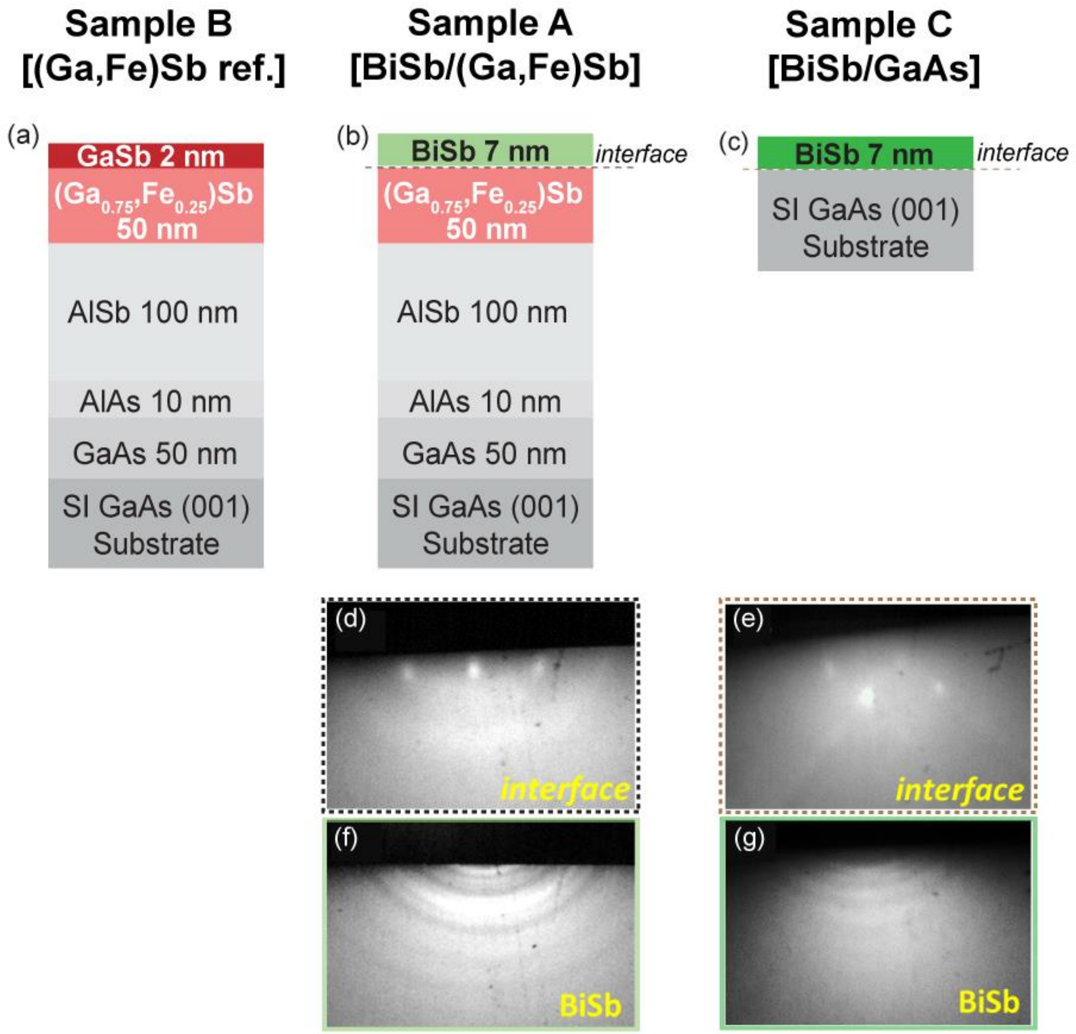

Figure S10. (a)–(c) Schematic illustration of the (001)-oriented sample structures of (a) (Ga,Fe)Sb reference sample (sample B), (b) BiSb/(Ga,Fe)Sb sample (sample A), and (c) BiSb/GaAs sample (sample C) grown on semi-insulating (SI) GaAs(001) substrates. (d) and (e) Spotty RHEED of the interface after etching (d) the GaSb cap layer of sample B [ref. (Ga,Fe)Sb] for the preparation of sample A [BiSb/(Ga,Fe)Sb] and (e) the GaAs oxidized surface in sample C [BiSb/GaAs], respectively. The black dotted line in (b) and (d) corresponds to the interface between BiSb and (Ga,Fe)Sb layer and the brown dotted line in (c) and (e) corresponds to the interface between BiSb and GaAs layer. (f) and (g) Ring RHEED patterns of BiSb during sputtering in sample A and sample C, respectively.

(b) <u>Conductivity measurements</u>

Next, we measured the temperature dependence of electrical conductivity $\sigma(T)$ of the

BiSb layer in sample A [BiSb/(Ga,Fe)Sb] and sample C [BiSb/GaAs]. Because Bi and Sb both belong to the same group-V elements, BiSb is free from doping effects due to off-stoichiometry issues and unintentional enhancement of conductivity is unlikely. Thus, the surface conductivity and bulk conductivity can be modeled using the surface and bulk parallel conduction model[S5] (see Sec 6). Figure S11 shows the measured temperature dependence of the normalized resistivity $\rho/\rho_{300\mathrm{K}}$, where ρ is the resistivity at different temperatures and $\rho_{300\mathrm{K}}$ is the resistivity at room temperature of the 7-nm-thick BiSb layer in sample C [BiSb/GaAs] and the whole layers [BiSb(7nm)/ GaFeSb(50nm)/ AlSb(100nm)/ AlAs(10nm)/ GaAs(50nm)] in sample A [BiSb/(Ga,Fe)Sb]. Note that parallel conduction in the buffer layers and substrates are negligible and thus we measure the conductance of the BiSb layers. Dashed lines are theoretical fitting by a parallel conduction model of BiSb. As shown in Fig. S11, **both samples show similar behavior with almost the same conductivity σ~ 1.8 × 10^5 (Ω^{-1}m^{-1}) [sample A] and 2 × 10^5 (Ω^{-1}m^{-1}) [sample C] at room temperature.** These large and similar conductivity values indicate the dominant contribution of the topological surface states (SSs) in the total conductivity of the BiSb layers (we will show this later by using Eq. (S5)). Moreover, the estimated value of the BiSb conductivity in our work is also in agreement with that (~ 2 × 10^5 Ω^{-1}m^{-1}) in ref S7.

Next, we determine the surface state contribution to the total conductivity, Γ, of the BiSb layer, defined by using the following equation

$$\Gamma = \frac{I_{\mathrm{Surface}}}{I_{\mathrm{Surface}} + I_{\mathrm{Bulk}}} = \frac{G_{\mathrm{S}}}{G}, \qquad \text{(S5)}$$

where I_{Surface} and I_{Bulk} are the currents flowing in the surface states and the bulk states, respectively. **We found that Γ is ~ 97% for both samples.** Moreover, the experimental data

and estimated Γ values in this study also agree with the results in Sec 6 (Fig. S8(a)), which showed temperature dependence of the ρ/ρ_{300K} of BiSb films with various thicknesses (t = 10 nm, 41 nm, and 92 nm). As shown in Sec 6 that Γ rapidly increases with decreasing the BiSb thickness and reaches nearly 90% for the samples thinner than 20 nm. This means that the topological surface state conduction in BiSb becomes dominant when the BiSb thickness $t \leq 10$ nm. We note that both the bulk and surface states are 2D in nature. Therefore, the "bulk" term in this work means trivial QW states (*i.e.* quantized bulk states). Because of the strong quantum size effect in BiSb, the "bulk" band gap (the energy separation between the first level of the trivial QW electron state and hole state) increases up to about 200 meV at t = 10 nm from our transport measurement[S7]. Also, very recently, Xing *et al.*[S9] successfully showed that BiSb is a topological insulator by observing quantum well states by scanning tunneling microscopy (STM) and detected a *clear bandgap ~ 0.39 eV (much larger than the 20 meV for bulk BiSb)* for *5.5-nm-thick BiSb, which is explained by the quantum confinement effect*[S5]. Furthermore, *they found that the Fermi level lies in the bandgap of BiSb*. Since we are using 7-nm-thick BiSb with Γ = ~97%, nearly all the current flows in the surface states of BiSb at room temperature. These results verify our conclusion that almost all the electrical transport occurs in the topological surface states of BiSb.

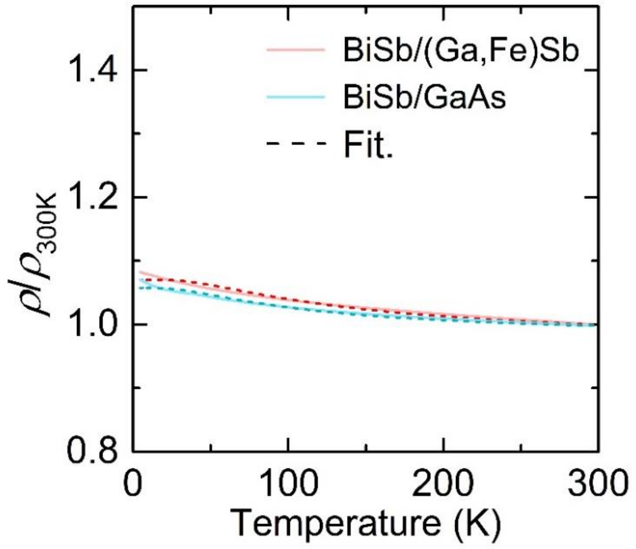

Figure S11. Temperature dependence of normalized resistivity ρ/ρ_{300K} in the BiSb/(Ga,Fe)Sb (sample A) and BiSb/GaAs (sample C). The dashed lines are fitting curves parallel conduction model (see Sec 6).

References

[S1] Smith. J., & Beljers, H.G. Ferromagnetic Resonance Absorption in, $BaFe_{12}O_{19}$, a Highly Anisotropic Crystal *Phillips Res. Rep.* **10**, 113 (1955).

[S2] Goel, S., Anh, L.D., Tu, N.T., Ohya, S., & Tanaka, M. In-plane to perpendicular magnetic anisotropy switching in heavily-Fe-doped ferromagnetic semiconductor (Ga,Fe)Sb with high Curie temperature. *Phys. Rev. Materials* **3**, 084417 (2019).

[S3] Rojas-Sánchez, J. C. *et al*. Spin pumping and inverse spin Hall effect in platinum: the essential role of spin-memory loss at metallic interfaces. *Phys. Rev. Lett.* **112**, 106602 (2014).

[S4] Fan, T., Khang, N. H. D., Nakano, S., & Hai, P. N. Ultrahigh efficient spin orbit torque magnetization switching in fully sputtered topological insulator and ferromagnet multilayers. *Sci. Rep.* **12**, 2998 (2022).

[S5] Xiao, S., Wei, D., & Jin, X. Bi (111) thin film with insulating interior but metallic surfaces. *Phys. Rev. Lett.* **109**, 166805 (2012).

[S6] Ueda, Y., Khang, N. H. D., Yao, K., & Hai, P. N. Epitaxial growth and characterization of $Bi_{1-x}Sb_x$ spin Hall thin films on GaAs(111)A substrates. *Appl. Phys. Lett.* **110**, 062401 (2017).

[S7] Fan, T., Tobah, M., Shirokura, T., Khang, N. H. D., & Hai, P. N. Crystal growth and characterization of topological insulator BiSb thin films by sputtering deposition on sapphire substrates. *Jpn. J. Appl. Phys.* **59**, 063001 (2020).

[S8] G. Landolt, *et al.*, Spin texture of Bi_2Se_3 thin films in the quantum tunneling limit. *Phys. Rev. Lett.* **112**, 057601 (2014).

[S9] Xing, S. *et al*. Epitaxial fabrication of topological Bi-Sb alloy films by surface alloying of Sb nanofilms. *Surf. Sci.* **714**, 121921 (2021).
